# Metalloproteinases and colorectal cancer. Correlation of gene expression and clinical-pathological parameters^1^

**DOI:** 10.1590/s0102-865020200070000007

**Published:** 2020-08-14

**Authors:** Sandra Regina Morini, Marcos Vinicius Denadai, Jaques Waisberg, Gaspar de Jesus Lopes, Delcio Matos, Sarhan Sydney Saad

**Affiliations:** I MD, Department of Pathological Anatomy, Hospital de Câncer de Barretos, Fundação Pio XII, Barretos-SP, Brazil. Acquisition and interpretation of data, technical procedures, statistical analysis, manuscript preparation and writing.; II MD, Department of Surgery, Hospital de Câncer de Barretos, Fundação Pio XII, Barretos-SP, Brazil. Acquisition and interpretation of data, technical procedures.; III PhD, Associate Professor, Department of Medicine, Universidade Federal de São Paulo (UNIFESP), Brazil. Interpretation of data, critical revision.; IV PhD, Full Professor, Department of Surgery, Faculdade de Medicina do ABC, Santo Andre-SP, Brazil. Interpretation of data, critical revision.; V PhD, Full Professor, Department of Surgery, UNIFESP, Sao Paulo-SP, Brazil. Scientific, intellectual, conception and design of the study.; VI PhD, Associate Professor, Department of Surgery, UNIFESP, Sao Paulo-SP, Brazil. Scientific, intellectual, conception and design of the study; critical revision; final approval.

**Keywords:** Metalloproteases, Colorectal Neoplasms, Extracellular Matrix

## Abstract

**Purpose:**

To analyze gene and protein expression of metalloproteinases 1, 2, 9, 11 and 16 and their correlation with clinicopathological variables in colorectal adenocarcinoma.

**Methods:**

A retrospective study of 114 patients with colorectal adenocarcinoma treated surgically in the period 2006 to 2008 in Hospital de Câncer de Barretos - Fundação Pio XII. The evaluation of gene expression was performed by RT-PCR, and protein by immunohistochemistry. The analysis of gene expression was classified as overexpressed genes and poorly expressed (fold change of approximately 2, p<0.05). The positivity of the markers in the immunohistochemical study was performed by semi-quantitative analysis. The tissue of TMA (Tissue Microarray) was done by two independent pathologists.

**Results:**

The gene expression validated by immuno - histochemical was MMP-1(p= 0.00 and 1.57 fold change) and MMP – 2 (p= 0.01 and – 1.84 to fold change) when correlated with the histological types mucinous and adenocarcinoma NOS, MMP9 (p=0.01 and fold change of 1.13) and MMP-16 (p=0.03 and 1.61 fold change) when compared with the histological types villous and adenocarcinoma NOS, MMP - 11 statistically significant in relation to male (p = 0.04 and 1.65 fold change).

**Conclusions:**

The MMPs 1, 2, 9, 11 and 16 gene and protein expression with statistical significance in at least one of the clinicopathological variables studied. Thus, we conclude that these MMPs have potential as a prognostic factor in colorectal adenocarcinoma.

## Introduction

Colorectal cancer (CRC) has a high incidence in the population. It was estimated that in the United States, in 2018, there would be 140.250 new cases of this disease, with the death of 50.630 patients^1^. In Brazil, according to the National Cancer Institute, for the year 2020, 20.520 new cases are expected in men and 20.470 in women. In 2017, there were 9.207 deaths of men and 9.660 of women resulting from this disease^2^.

The study of colorectal cancer carcinogenesis is of paramount importance for us to understand how the neoplasm develops and its progression, which can result in the formation of local and distant metastases. It is a complex process with many stages characterized by changes in the quantity or activity of proteins that regulate cell proliferation, differentiation and survival, a process mediated by genetic and epigenetic mechanisms.

Different processes involving carcinogenesis have been the subject of research. One of them is the relationship between tumor cells and adjacent normal tissue that includes proteolytic enzymes^3^. The cellular invasion of tissue may occur during various stages, either physiological or pathological. There are barriers that oppose the motion of the tissue invasion, like basement membranes, the stromal matrix and the junction between cells. It is believed that a common mechanism for breaking these barriers is the action of proteolytic enzymes, which act on the type of tissue involved and the conditions found in the extracellular matrix (ECM)^4^ ^,^ ^5^.

Metalloproteinases (MMPs) form a group of enzymes (endopeptidases) that are responsible for the degradation of ECM and basement membranes. Several studies have been conducted to clarify and improve our knowledge about MMPs^6^ ^–^ ^8^. This group of enzymes plays an important role in tumor progression by enabling the invasion of blood and lymph vessels that are close to the tumor. The degradation of ECM components by MMPs contributes to the removal of physical barriers to cancer. This occurs at various stages of disease progression, including local growth, neoangiogenesis, invasion and extravasation. Therefore, several MMPs have been involved with tumor progression, both in its invasive behavior and in its ability to metastasize. In most human cancers, the expression and activity of metalloproteinases are high when compared to normal tissue, and the same is true for colorectal cancer^3^ ^,^ ^9^.

MMP-1 is a typical membrane enzyme with collagen type I, II and III VII, VIII, X, XI, gelatins, fibronectin and others as substrate^10^. MMP-2 and MMP-9 are essential for tumor dissemination, because in addition to playing an important role in the development of invasive processes, they degrade the components of basement membranes, like type IV collagen^11^. MMP-9, known as gelatinase B, specifically in colorectal cancer, appears as a protease that regulates tumor cell growth, mobility and survival. The complex interaction between MMP-9 activity, regions of the active membrane and signaling by adhesion molecules regulates the migration of tumor cells, invasion and metastases^8^ ^,^ ^12^. MMP-11 is highly expressed in colonic carcinoma, as well as in several neoplasms^13^. The increased expression of MMP-11, both in epithelial and tumor stromal cells, suggests that neoplastic cells diffusely produce factors that induce fibroblasts to synthesize this protease. MMP-16 (MT3-MMP) has high expression linked to tumor invasion in several types of cancer^14^, but there are no reports of MMP-16 positivity in colorectal cancer.

The best method of prognostic evaluation for colorectal tumors is still the anatomic-pathological report, which enables us to assess the grade of invasion in the organ wall and the lymphatic system. However, we are also looking for other markers that can assist in this prognostic evaluation. In most human cancers, MMP expression and activity are high when compared to normal tissue. This has also been demonstrated in CCR, and MMPs can be used as tumor markers to prevent tumor growth, invasion and metastasis^6^ ^,^ ^9^ ^,^ ^15^.

The objective of this study is to analyze gene and protein expression of metalloproteinases 1, 2, 9, 11 and 16 and their correlation with clinicopathological variables in colorectal adenocarcinoma.

## Methods

This study was carried out with 125 patients at Hospital de Câncer de Barretos - Fundação PIO XII, from 2006 to 2008. Clinical data were collected from these patients’ medical records. To determine the diagnosis and the staging, the following were used: biopsy of the colorectal tumor, imaging exams, intraoperative findings and anatomic-pathological report of the surgical specimen. It was based on the TMN classification adopted by the American Joint Committee on Cancer^16^.

The following exclusion criteria were considered: patients with familial polyposis and histological types other than carcinoma. We also excluded cases in which adequate samples of RNA were not obtained in the extraction (11 cases out of a sample of 125).

Of the 114 patients, 51 (44.7%) were women and 63 men (55.3%) with ages ranging from 24 to 85 years, with an average of 54.5 years. The data related to the neoplasm and its histopathological study are shown in Table 1. There were no cases of Grade IV undifferentiated neoplasms.

**Table 1:** Characteristics of neoplasms in terms of location, staging, histological type, grade of cell differentiation, perineural and angiolymphatic invasion.

	N.	%
Colon	82	71.9
Rectum	32	28.1
Stage I	25	21.9
Stage II	39	34.2
Stage III	35	30.7
Stage IV	15	13.2
NOS adenocarcinoma*	81	70.0
Mucinous adenocarcinoma	18	15.9
Villous adenocarcinoma	15	13.1
Well differentiated - Grade I	9	7.9
Moderately differentiated - Grade II	92	80.7
Poorly differentiated - Grade III	13	11.4
Angiolymphatic invasion	23	20.2
Perineural infiltration	8	7.0
Peritumoral inflammatory infiltrate	83	72.8

* NOS - Not Otherwise Specified

The samples were collected and frozen immediately after the surgical specimens were removed. One to four fragments of tumor tissue were collected. They measured 0.5 to 1.0 cm³ and were stored at a temperature of −80°C. For the extraction of RNA, the tumor tissue was placed in microtubes (2ml) containing 500 µl of Trizol (Invitrogen) obtained by the RNeasy Mini Kit (Qiagen, cat. No. 74104). The RT-PCR reaction was performed using the SuperScript III FirstStrand Synthesis SuperMix kit (Invitrogen, USA). The synthesis of cDNA was made from 1 µg of total RNA extracted from tumor tissue using the SuperScriptTMIII First-Strand Synthesis System for RT-PCR kit (Invitrogen Cat. No. 18080051).

Of the 125 samples selected for the study, 11 were contaminated by genomic DNA and were therefore excluded. The cDNA obtained through the total RNA of the tumor samples was added to the plate with 84 genes of the SYBR Green Master Mix system. Expression levels were measured for each specific gene and optimized for simultaneous use on the PCR Array platform. In this study, the Extracellular Matrix and Adhesion Molecules PCR Array (PAHS-013) (SABioscience, Qiagen, Valencia, California, USA) was investigated. The validation of gene expression was done through immunohistochemical study. The slides and histological data were reviewed by at least two pathologists. The most representative slides of the tumor were selected, and these areas were marked for the TMA (Tissue Micro-Array). The primary monoclonal antibodies we used were from the ABCM INC manufacturer: anti-MMP-1 (reference: ab52631 - clone EP1247Y); anti-MMP-2 (reference: ab52756 - polyclonal); anti-MMP9 (reference: ab76003- clone EP1254); anti-MMP-11 (ref ab53143-polyclonal); anti-MMP-16 (reference: ab73877- polyclonal). The standardized assessment of all markers was based on the “Quick score” *Q = P x I* (17) in which P corresponds to the percentage of diffuse and uniformly positive epithelial cells: 0 – negative; 1 - <25% of positive epithelial cells in the cytoplasm; 2 − 26-50%; 3 -> 50%; and I the intensity of staining: 1 – light; 2 – moderate and 3 – intense. The expression was considered weak positive (1) in cases with a score of 0 to 5 and strong positive (2) in a score of 6 or more^18^.

### Statistical analysis

The analyses were performed using the SPSS – Statistical Package for Social Sciences (v15.0). To compare qualitative variables, that is, frequencies and proportions, we used Fisher's exact test. To verify whether the data were showing normal distribution, we used the Shapiro-Wilk test. To compare quantitative data, we used the Mann-Whitney U test whenever necessary. The value of statistical significance was set at 5%, or p <0.05.

## Results

Of the 84 genes, low-expression and over-expression genes were selected. When analyzing the expression of these genes, we considered a significance level of p <0.05 and a fold change of approximately 2.

The expression of MMP-1 was significantly related when comparing the mucinous histological type with NOS adenocarcinoma (p = 0.00 and fold change of 1.57) and with the presence of peritumoral inflammatory infiltrate (p = 0.01 and fold change of −1.84). Similarly, MMP-2 occurred with mucinous and NOS adenocarcinoma histological types (p = 0.01 and fold change of 2.17) and peritumoral inflammatory infiltrate (p = 0.04 and fold change of −1.2). This MMP was also significantly related when comparing patients over 60 years old with those under 60 (p = 0.04 and fold change of −2.11).

MMP-9 and MMP-16 show greater expression with villous histological type versus NOS adenocarcinoma (p = 0.01 and fold change of 1.13 and p = 0.03 and fold change of 1.61). MMP-11 has shown statistical difference in its expression in males (p = 0.04 and fold change of 1.65) (Table 2).

**Table 2:** Gene expression of MMPs and respective validations by IH* and its correlation with clinical and anatomic-pathological characteristics.

Genes	Variable	p	Fold change	Validation by IH*	p
MMP 1	Mucinous *vs*. NOS**	0.04	1.57	yes	0.001
	Inflammatory infiltrate	0.05	-1.84	no	0.001
MMP 2	Mucinous *vs*. NOS**	0.01	2.17	yes	0.001
	Inflammatory infiltrate	0.04	-1.2	no	1.000
	Age (> 60y *vs*. <60y) ***	0.03	-2.11	no	0.001
MMP 9	Villous *vs*. NOS	0.01	1.13	yes	0.001
MMP 11	Gender: M *vs*. F****	0.04	1.65	yes	0.001
MMP 16	Villous *vs*. NOS	0.03	1.61	Yes	0.001

* Immunohistochemistry.

** Not otherwise specified adenocarcinoma.

*** Over 60 years old *vs*. under 60 years old.

**** Male *vs*. female.

## Discussion

The cellular invasion of a tissue can occur during several stages, either physiological or pathological. There are barriers that oppose the movement of tissue invasion, like basement membranes, stromal matrix and the junction between cells. A common mechanism for breaking these barriers is believed to be the action of proteolytic enzymes. Evidence suggests that proteinases, specifically MMPs, are involved in this process and that these proteins can be used as prognostic markers of colorectal cancer^8^ ^,^ ^15^ ^,^ ^18^.

PCR Arrays are reliable tools to analyze the expression of a panel of genes that are specific to a certain pathology, offering high sensitivity and a wide dynamic range. This method analyzes the expression of a group of proteins involved in the progression and dissemination of colorectal adenocarcinoma in its various stages^19^ ^–^ ^21^.

MMP-1 is a collagenase that degrades collagens I, II and III. The lysis of these proteins, which are primary components of the stroma of the GI tract, enables the invasion of neoplastic cells into the intestinal wall. Furthermore, the increased expression of MMP-1 shows a statistically significant correlation when lymph node and metachronous metastases are present, suggesting that this enzyme plays a part in tumor dissemination^15^ ^,^ ^17^. In the present study, both the gene and protein expression of MMP-1 have shown a statistical correlation with the histological type, with greater expression in mucinous types with signet ring cells than in the NOS adenocarcinoma type, suggesting that it is related to malignancies of worse prognosis.

MMP-2, called gelatinase A, degrades, in addition to collagen IV, other types of this protein, like V, VII, and X, in addition to fibronectin, laminin and elastin. In studies comparing the expression of MMP-2 in CRC and the clinical-pathological variables, there was a statistical significance of strong expression of this enzyme in cases of stage III and IV, tumor size and venous invasion, lymph node metastasis and distant metastasis. This suggests that, even at an early stage, MMP-2 expression is correlated with unfavorable prognostic factors^11^ ^,^ ^22^ ^–^ ^25^. In our study, there was a correlation between MMP-2 gene and protein expressions with the mucinous histological type with signet ring cells and NOS adenocarcinoma. Our results, like others in the literature, have shown that MMP2 can have great potential as a prognostic marker for CRC.

MMP-9, known as gelatinase B, promotes the degradation of an important component of the basement membrane, which is collagen IV. It is therefore crucial in the invasion of malignant tumors through the proteolysis of the ECM with progression and metastasis in CRC^26^. Several studies in the literature have shown the increased expression of MMP-9 in CRC when compared to clinical-pathological variables like stage III and IV, lymph node metastases, distant metastases, peritumoral inflammatory infiltrate and grades of cell differentiation II and III. All these studies suggest that the strong expression of MMP-9 is related to a worse prognosis^10^ ^,^ ^11^ ^,^ ^24^ ^,^ ^27^ ^–^ ^31^. In our study, MMP-9 expression was significantly more frequent in relation to the villous histological type, which has a better prognosis than NOS adenocarcinoma^32^.

MMP-11 (stromelysin 3) plays an important role in tissue remodeling and is seen in tumor growth and metastases. Authors have associated the increase in MMP-11 with a poor prognosis in breast^33^ and gastric^34^ ^,^ ^35^ cancer. There are few reports of MMP-11 expression in CRC; some only observed increased expression at the edge of the tumor and location of the neoplasm^36^, in colorectal cancer in relation to non-tumor tissue and in the primary tumor in relation to metastasis^13^ ^,^ ^37^. In our study, there was a correlation of this enzyme only with the male gender, which coincides with data from the literature.

In the literature, there is no study correlating the expression of MMP16 with CRC. In the present study, there was a statistically significant correlation between the expression of MMP-16 and CRC when greater expression was observed in villous types, which have a better prognosis than the NOS adenocarcinoma type. This data may open a perspective for further investigations on MMP-16 in CRC.

## Conclusions

The expressions of MMP-1 and MMP-2 have shown a statistical correlation to the mucinous histological type with signet ring cells when compared to NOS adenocarcinoma. The expressions of MMP-9 and MMP-16 have shown a statistical relationship with the villous histological type in comparison to NOS adenocarcinoma. MMP-11 has shown expression with statistical significance in relation to the male gender. MMPs 1, 2, 9, 11 and 16 are related to at least one clinical-pathological variable, which corresponds to prognostic factors of CRC. The conclusion is that these enzymes are potential prognostic markers for this type of tumor.
